# Differences in the Access to Sterilization between Women Living and Not Living with HIV: Results from the GENIH Study, Brazil

**DOI:** 10.1371/journal.pone.0164887

**Published:** 2016-11-03

**Authors:** Regina Maria Barbosa, Cristiane da Silva Cabral, Tania di Giacomo do Lago, Adriana de Araujo Pinho

**Affiliations:** 1 Núcleo de Estudos da População Elza Berquó –Universidade Estadual de Campinas–Campinas, Brazil; 2 Centro de Referêcia e Treinamento em DST/AIDS–Secretaria de Estado da Saúde de São Paulo–São Paulo, Brazil; 3 Departamento de Saúde Materno-Infantil–Faculdade de Saúde Pública–Universidade de São Paulo–São Paulo, Brazil; 4 Instituto de Saúde–Secretaria de Estado da Saúde de São Paulo–São Paulo, Brazil; 5 Laboratório de Educação em Ambiente e Saúde–Instituto Oswaldo Cruz–Fiocruz–Rio de Janeiro, Brazil; Universidade Estadual de Maringa, BRAZIL

## Abstract

**Background:**

In many countries, young women of reproductive age have been especially affected by the HIV epidemic, which have fostered research to better understand how HIV infection influences and shapes women´s fertility and reproductive and sexual decisions. In Brazil, few studies have focused on the impact of the HIV epidemic on contraceptive choices among women living with HIV (WLHIV).

**Objective:**

This study evaluates the impact HIV infection may have in the access to female sterilization in Brazil, using a time-to-event analysis.

**Methods:**

A cross-sectional quantitative study (GENIH study) was conducted between February 2013 and April 2014 in the city of São Paulo, comparing two probabilistic samples of 975 WLHIV and 1,003 women not living with HIV (WNLHIV) aged 18 to 49. Sexual and reproductive data was collected retrospectively in order to reconstruct women's reproductive trajectories. Given the objectives of this study, the analysis was restricted to women with parity one or more and, in case of WLHIV, to those sterilized after HIV diagnosis and not infected through vertical transmission. The final sample analysis included 683 WNLHIV and 690 WLHIV. A series of multivariable-adjusted Cox models estimated the probability of being sterilized after HIV diagnosis, compared with WNLHIV. Models were adjusted for schooling, race/color, and stratified by parity at last delivery (1–2, 3+). Hazard ratios were calculated for female sterilization, and separately for interval and postpartum procedures (performed in conjunction with caesarean section or immediately after vaginal delivery). Additionally, information regarding unmet demand for female sterilization was also explored.

**Findings:**

No statistical difference in the overall risk of sterilization between WLHIV and WNLHIV in the two parity-related groups is observed: HR = 0.88 (0.54–1.43) and 0.94 (0.69–1.29), respectively, among women with 1–2 children and those with three and more. However, significant differences regarding the impact of HIV infection at sterilization are observed depending on the timing and the type of sterilization procedure. The probability of obtaining an interval sterilization is significantly lower for WLHIV compared to those not living with HIV. The reverse occurs regarding postpartum sterilization. Although sterilization is mainly performed in conjunction with caesarean section in Brazil, it is evident that caesarean sections are not the sole factor increasing the risk of sterilization among WLHIV.

**Conclusion:**

The results indicate barriers in the access to services offering interval sterilization for WLHIV and certain facilitation in obtaining the procedure in conjunction with caesarean section. Health policy makers at local and national levels should promote institutional changes in order to facilitate access to interval sterilization and to confront the sensitive discussion of WLHIV’s eligibility for postpartum sterilization. It is also urgent to increase access to a wider range of contraceptive methods for WLHIV and promote dual method protection strategies. Moreover, since condom use may decrease in the future in the context of the preventive effect of antiretroviral therapy, promoting dual methods will expand the choices regarding the reproductive rights of women living with HIV.

## Introduction

In many countries, young women of reproductive age have been especially affected by the HIV epidemic [1], which have fostered research to better understand how HIV infection influences and shapes women´s fertility and reproductive and sexual decisions.

Several studies carried out mostly before, but also after the implementation of the AIDS Clinical Trials Group (ACTG) Protocol 076—a clinical trial which showed the efficacy and safety of AZT in reducing mother-to-child transmission (MTCT) [2]—showed divergent findings regarding reproductive outcomes among WLHIV. Higher abortion rates, a decline in fertility rates, and an increase in voluntary sterilization rates were observed by some studies [3–7]. However, others [8–10] observed no differences or smaller rates in the same outcomes.

The lack of clear evidence on how reproductive preferences and practices of women living with HIV differ from other women poses us a number of unanswered questions and different issues. The existence of laws allowing pregnancy interruption, attitudes from health professionals in relation to sterilization and abortion, access to contraceptive methods and health services as well as to HIV prevention and treatment are strong enough elements to shape women’s reproductive decisions differently according to their position and interconnection within each society. In this sense, studies focusing in specific contexts still need to deepen the knowledge about HIV infection and the resulting reproductive practices and decision, as is the case of female sterilization.

Total fertility rate dropped rapidly in Brazil, falling from 6.28 children per woman in 1960 to 4.35 in 1980, 2.38 in 2000, and below the replacement level in 2010 [11]. The increased use of modern contraception is considered one of the main factors associated with this drop [12–13]. Female sterilization has become the focus of debates on reproductive rights in the past few decades due to an increased trend in its use, followed by a decrease after the passing of Law No. 9263—also known as the Family Planning Law. The law and the regulations for its implementation, issued in 1997 and 1999 by the Federal Government [14], legalized the practice of sterilization and established explicit guidelines for its use.

For the first time, the procedure was authorized to be funded by the Brazilian Public Health System (Sistema Único de Saúde—SUS). On one hand, men and women who are at least 25 years of age, or those who have at least two living children, became eligible to request the procedure. On the other hand, the law prohibited postpartum sterilizations as an attempt to reduce the number of cesarean sections for sterilization purposes. Exceptions are made in the case of an existing illness and for those to whom a second surgery, or additional exposure to anesthesia, would represent a major health risk. The law also required a minimum 60-day waiting period between the date the request is made and the actual surgery; in addition, participation in family planning education groups was encouraged.

Female sterilization rates fell among married and cohabiting women between 15–44 years of age, from 38.5% in 1996 to 25.9% in 2006, while vasectomies increased from 2.8 to 5.1% [15–16]. The prevalence of hormonal oral contraceptive experienced a small increase, from 23.1% to 27.4%, and the use of condoms increased significantly during the period, from 4.6 in 1996 to 13% in 2006. The use of other methods remained relatively stable, around 10%, and the non-use of contraception decreased from 22.1% in 1996 to 18.4% in 2006 [16].

The prevalence of female sterilization among the general population in Brazil varies widely according to region of the country. Women living in the North, Central-West and Northeast regions are 2.5 times more likely to be sterilized than those living in the Southeast and South regions [16]. Despite the legal impediments established by the law, female sterilization is still carried out in conjunction with caesarean section, which has remained the most prevalent childbirth method [17]. The procedure is more prevalent among women in older cohorts, with parity higher than two, with less schooling and among black women [13;15–16]. Women with less schooling tend to be sterilized without having used another contraceptive method and without reaching the ideal number of children [16].

On the other hand, the rapid increase in the number of AIDS cases since the 1980s in Brazil and elsewhere raised a substantial change in sexual practices and brought the use of condoms to the center of the debate on STI prevention [18–19]. The HIV epidemic also affected reproductive practices and choices, especially among women living with HIV [20–22].

Brazil has approximately 280,000 officially documented cases of AIDS among women, the vast majority of whom are of reproductive age [23]. Antiretroviral therapy (ART) is widely available, and new cases of AIDS are decreasing rapidly among children under 1 year of age as a result of the prevention of mother-to-child transmission (PMTCT). The majority of WLHIV have low levels of education and income [23–24]. Additionally, studies have emphasized that women’s vulnerability to HIV is directly associated with gender inequality, which reduces their power to negotiate condom use and other prevention practices with their partners [18–19]. Precarious access to family planning methods, lack of information about how to prevent pregnancy and lack of access to safe abortion complete the scenario in which WLHIV make their reproductive decisions and choices in Brazil [22;25–28].

In Brazil, studies have suggested higher prevalence of sterilization among HIV-positive women compared to their HIV-negative or not HIV tested counterparts [29–30]. One of them also indicated women’s selective access to sterilization depending on the health care professionals' opinion on sterilization and their preconceptions concerning the reproductive rights of WLHIV [20]. Information regarding the factors associated with female sterilization among WLHIV in Brazil derived from only two studies. The procedure is more prevalent among women with parity higher than two [21;30] or who had a child after HIV diagnosis or were sexually active after HIV diagnosis [21]. Its prevalence is similar among women with some level of education, and the great majority of women sterilized after HIV diagnosis had the procedure performed in postpartum at the time of caesarean section [21;30].

Such evidence, however, generated by a small number of studies conducted in Brazil, mainly refers to the period prior to PMTCT and the Family Planning Law, or at most to the beginning of its implementation. To the best our knowledge, none of the studies have considered in their analysis the women’s age at sterilization or have provided estimates of the probability of obtaining female sterilization separately for postpartum and interval sterilization.

In view of these gaps, the objective of this study is to evaluate the impact HIV infection may have in the access to female sterilization in Brazil, using a time-to-event analysis. Moreover, it compares the probability of obtaining female sterilization between WLHIV and WNLHIV, separately for postpartum and interval sterilization.

## Materials and Methods

### Data and study sample

The primary data used for this analysis comes from a cross-sectional quantitative study based on a life course perspective [31], named the GENIH study. Its main goal was to investigate aspects of sexual and reproductive health of women living with HIV (WLHIV) and compare them with those of women not living with HIV–WNLHIV (women with HIV-seronegative results or unknown HIV status).

The study was conducted between February 2013 and April 2014 in the city of São Paulo, comparing two probabilistic samples of 975 WLHIV and 1,003 WNLHIV aged between 18 and 49, users of public health services. São Paulo is the largest city in Latin America (about 12 million inhabitants) and concentrates one third of the total accumulated cases of AIDS of Brazil.

Participants completed an in-person computer-based structured interview, which retrospectively investigates reproductive and sexual events and the timing of their occurrence.

Participants were selected using a multistage probability sample design of women’s public health care service users (WNLHIV) and WLHIV users of specialized HIV health services in the city of São Paulo. The WLHIV sample included all 18 HIV public health services that comprise the health care reference network for people living with HIV in the city of São Paulo. This network accounts for 95% of the care provided to women living with HIV. These units constituted the strata and the sample of 1,000 women was distributed proportionally to the size of each health unit.

The WNLHIV sample was also selected using multistage probability sampling. The first stage comprised strata constituted by all five Departments of Health within the municipality of São Paulo and the sample of 1,000 women was distributed proportionally to the size of these regions. The second stage included 38 primary health care services selected with probability proportional to size, from a total of 442 services that comprise the basic health network within SUS (Sistema Único de Saúde) in the city of São Paulo. In each unit, women were randomly selected (systematic sampling) from the list of daily appointments. We oversampled the estimated number of interviews by 25% as a safety margin for possible losses or refusal. The refusal rates for participation in the study in both groups were about 26%.

In each health unit, the field team was comprised by one supervisor and two or three interviewers. All of them were women, hold a degree in health or social sciences, and received a 40-hour training course.

The qualifying women were invited by field supervisors to participate in the study. The objectives of the investigation and its procedures were explained, and those eligible and who agreed to participate in the study were directed to a private room where the informed consent was applied. After signing it, a social-behavioral electronic questionnaire was administered with the aid of a netbook. The electronic questionnaires were generated using the software QDS^TM^ (Questionnaire Development System, by New Research Company) and pretested.

Given the objectives of this study, the analysis was restricted to women with parity one and more (n = 1448), since sterilization among nulliparous women is extremely rare. WLHIV who were sterilized before diagnosis (n = 61) and those who were infected with HIV through vertical transmission (n = 14) were also excluded from the analysis. Women infected through vertical transmission are younger (younger than 24 years of age) and their sexual and reproductive trajectories have been shaped since the beginning by the presence of the HIV virus. This fact gives to their trajectories certain distinctive features that deserve specific analysis, which is not the objective of this study. The final sample analysis included 683 WNLHIV and 690 WLHIV.

### Statistical analysis

Information on female sterilization is used as the dependent variable, considering the age at sterilization. Women were directly asked if sterilization was performed at a time not related to childbirth (interval sterilization) or at delivery (postpartum sterilization): in conjunction with a caesarean section or immediately after childbirth. Therefore, it was also possible to separately analyze postpartum sterilization carried out during caesarean delivery, which is one of the contributions of this study.

Considering results from previous studies [15;16;30;32], the following independent variables were used to analyze the prevalence and the risk of obtaining female sterilization among WLHIV and WNLHIV: schooling at the time of the interview (incomplete elementary school and lower, complete elementary school and higher), race/color (brown “parda”, black “preta”, white “branca”), and parity at last delivery (1–2, 3+).

For the purpose of this study, the event (female sterilization) was analyzed as follows:

First, we calculated for each group (WLHIV and WNLHIV) the frequency of female sterilization by parity, educational level, race/ethnicity and type of last delivery (cesarean section or vaginal). We also calculated the mean and median age at sterilization for both groups and the frequency of each sterilization procedure, interval or postpartum sterilization (in conjunction with a caesarean section or immediately after vaginal delivery).

Secondly, a series of models estimated the probability of being sterilized at certain age once HIV is diagnosed, compared to the risk of being sterilized at the same age in the absence of HIV diagnosis (WNLHIV), using a time-to-event analysis. As having more than two children increases the prevalence of female sterilization, both for WLHIV [21] and WNLHIV [16], the analyses were stratified by parity. For these analyses, we fitted multivariable-adjusted Cox models to calculate hazard ratios and 95% confidence intervals for undergoing the procedure. The hazard function assesses the risk at a particular moment that an individual, who has not yet done so, will experience the target event: female sterilization. The numerator of the hazard function represents the conditional probability that the event will occur in a specific interval given that it has not occurred before, and the denominator represents the interval width.

The data used to calculate the time (in years) of exposure to the risk of sterilization derived from information regarding the respondents’ age at sterilization, at HIV diagnosis, and at the time of the interview. The time of exposure to the risk of sterilization is equal to the interval between age 15 (when all women in the sample had no children) and the age by the time of the interview (right censored data) or the age of the event (female sterilization). In cases where no sexual intercourse was reported in the 12 months preceding the interview, women who had not had female sterilization were right censured at the time of the last sexual intercourse, since they were no longer at risk of getting pregnant.

The results of four analyses are shown and refer to: 1) the overall risk of getting sterilized irrespectively of it being an interval or postpartum procedure. All observations in both samples were included in this model; 2) the risk of having an interval sterilization. Observations related to postpartum sterilization were excluded in this model; 3) the risk of having a postpartum sterilization–both through cesarean section or immediately after vaginal delivery. In this model, only WLHIV who gave birth after HIV diagnosis were considered; and 4) the risk of having an sterilization carried out in conjunction with a caesarean section. In this model, only women who gave birth through cesarean section were considered in both groups.

Each analysis consisted of two models that took into account the parity of women (stratification variable), their level of schooling at the time of the interview and race/color (controlling variables). After adjustment for each final model, we tested the proportional hazard assumption of all models. The regression tables show the exponential of coefficients, known as hazard ratios. A set of graphs illustrates the cumulative probabilities of being sterilized in each model.

Lastly, we explored information regarding the demand for female sterilization, which is addressed in terms of the desire to have it carried out in the past (unmet demand) as well as preferences as to when and how to undergo the procedure: whether immediately, without having to wait to give birth; at the next childbirth; or at another time, or none of the above. In order to compare unmet demand among WLHIV and WNLHIV, we carried out a binomial logistic regression adjusted by age and parity.

All analyses were conducted using SPSS 20.0 and STATA 14.0 and have taken into account the complex sampling design of the study. The project was approved by the Research Ethics Committee of the Centro de Referência e Treinamento em DST/AIDS de São Paulo (under N. 022/2011), as well as by the ethics committees of other institutions involved, as follows: Instituto de Infectologia do Emílio Ribas (under N. 11712112.6.0000.5375), Secretaria Municipal de Saúde de São Paulo (under N 0043/12) and Universidade Federal de São Paulo (under N. 11712112.6.0000.5375). All interviewees signed a written informed consent term and were advised that they could refuse to participate in the survey at any time.

## Results

### Sociodemographic profile and sterilization situation

Among women with at least one child, 19.6% of WLHIV had ended their reproductive trajectory through sterilization after HIV diagnosis, compared to 16% of WNLHIV. Postpartum sterilization is the most frequent procedure used by both groups, but it is higher among WLHIV (16.5%) compared to WNLHIV (10.1%). The opposite is observed regarding interval sterilization, which is higher among WNLHIV (5.6%) compared to WLHIV (3.2%). (Table 1).

**Table 1:** Distribution of WLHIV and WNLHIV and proportion of sterilization by education, color/race, parity, type of delivery and age at sterilization. São Paulo, 2013–2014.

		Group				
	WLHIV			WNLHIV		
	N^μ^	% of women	% of sterilization	N^μ^	% of women	% of sterilization
**Total** ^**§**^	690	** **		683		
Sterilized ^£^	131	19.6%	-	112	16.0%	-
During a caesarean section	97	15.4%	-	65	9.2%	-
Immediately after vaginal delivery	8	1.1%	-	6	0.9%	-
Interval sterilization	23	3.2%	-	38	5.6%	
Not sterilized	559	80.4%	-	571	84.0%	-
Education						
Incomplete elementary and under	389	56.1%	19.0%	360	52.7%	21.9%
Complete elementary school	301	43.9%	15.2%	323	47.3%	10.2%
Color/Race						
White (“branca”)	278	39.7%	16.8%	262	38.5%	14.5%
Black (“preta”)	126	19.2%	24.4%	64	9.4%	23.4%
Brown (“parda”)/others	284	41.1%	15.2%	357	52.1%	16.5%
Parity						
1–2	464	68.0%	10.1%	490	72.4%	8.8%
3+	226	32.0%	31.1%	193	27.6%	35.8%
Type of delivery						
Caesarean	278	41.7%	38.8%	293	42.1%	24.9%
Vaginal	99	13.5%	15.2%	389	57.9%	10.0%
No delivery after HIV diagnosis	310	44.8%	2.2%	na	na	na
Age at sterilization, mean (median)	131	31.1 (31)		112	28.5 (28)	

^μ^ Totals may differ due to missing answers

§Women with parity = 0 for both groups, or infected by vertical transmission were excluded from this analysis

£ among WNLHIV proportion refers only to women sterilized after HIV diagnosis.

Information on educational attainment indicates that slightly more than half of the sample in both groups had not completed elementary school, corresponding to the highest proportion of sterilization events (19.0% among WLHIV and 21.9% among WNLHIV). However, it is noteworthy that the proportion of sterilization among women with more schooling is higher among WLHIV compared to WNLHIV (15.2% versus 10.2%).

In terms of color/race, the minority of both samples is composed of black women, although among WLHIV this proportion is significantly higher (19.2% among WLHIV versus 9.4% among WNLHIV). The proportion of sterilization is higher among black women in both groups (23.4% among WLHIV versus 24.4% among WNLHIV) compared to white and brown (parda).

In terms of parity, the great majority of both groups have had one or two children (68% among WLHIV versus 72.4% among WNLHIV), corresponding to the lower proportion of sterilization procedures. It is noteworthy that this proportion is slightly higher among WLHIV, 10.1%, compared to 8.8% among WNLHIV, and the opposite occurs among women with three or more children (31.1% versus 35.8%, respectively).

Caesarean section at the last delivery was carried out by 42.1% of WNLHIV. Among WLHIV, 41.7% experienced caesarean sections and 44.8% had no delivery after HIV diagnosis. The percentage of sterilization among women who had caesarean deliveries is much higher than among women who had vaginal deliveries in both groups, but much higher among women living with HIV: 38.8%, compared to 24.9% among WNLHIV. The proportion of the event among those who did not give birth after the HIV diagnosis is extremely low, 2.2%. The mean age at sterilization is higher among WLHIV, 31.5 years, compared to 28.5 among WNLHIV.

### Hazard models for female sterilization

The first hazard model includes all women with parity >0 and indicates no significant differences in the risk of female sterilization between WLHIV and WNLHIV, both for those with parity 1–2 as well as 3 or more (HR = 0.88; p = 0.595 and HR = 0.94; p = 0.698, respectively) (Table 2). Women who did not complete elementary school were two times more likely (p = 0.038) to have had female sterilization among those with lower parity. Black women with one or two children were almost three times more likely (p = 0.013) to have had the procedure among those with the same parity.

**Table 2:** Hazard ratios for female sterilization by HIV status, education and color/race, and stratified by parity. São Paulo, 2013–14.

Characteristic	HR (IC95%)	
	1–2 children	3+ children
Group		
WLHIV	0.88 (0.54–1.43)	0.94 (0.69–1.29)
WNLHIV (ref)	-	-
Education		
Incomplete elementary school and lower	1.66 (1.05–2.64)*	1.01 (0.68–1.48)
Complete elementary school and higher (ref)	-	-
Color/Race		
Brown (“parda”)	1.51 (0.93–2.45)	0.86 (0.60–1.25)
Black (“preta”)	2.86 (1.49–5.46)**	0.95 (0.57–1.58)
White (“branca”) (ref)	-	-

* p<0.05

** p<0.005; p<0.000.

**Fig 1:**
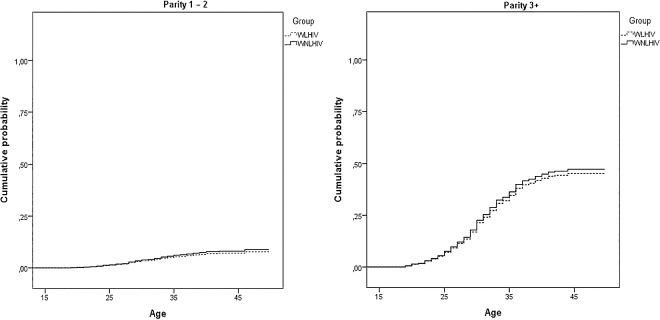
Cumulative probability of sterilization from models in Table 2 for women living and not living with HIV. São Paulo, 2013–14.

The cumulative probability of having had sterilization is much higher among women with three or more children in both groups and increases with age, until age 40, and then remains relatively stable (Fig 1).

The second hazard model was estimated only for observations related to interval sterilization and suggests that the risk of female sterilization is 62% lower [(0.38–1)*100] among WLHIV compared to WNLHIV with a parity of 3 or more (p = 0.011) and 56% lower among those with 1–2 children (p = 0.072), although the last association was only marginally significant (Table 3).

**Table 3:** Hazard ratios for interval sterilization by HIV status, education and color/race, and stratified by parity. São Paulo, 2013–14.

Characteristics	HR (IC95%)	
	1–2 children	3+ children
Group		
WLHIV	0.44 (0.18–1.08)	0.38 (0.18–0.80)*
WNLHIV (ref)	-	-
Education		
Incomplete elementary school and lower	2.50 (1.05–5.94)*	1.07 (0.48–2.39)
Complete elementary school and higher (ref)	-	-
Color/Race		
Brown (“parda)	1.36 (0.56–3.35)	1.16 (0.49–2.74)
Black (“preta”)	4.58 (1.39–15.1)*	1.29 (0.49–3.38)
White (“branca”) (ref)	-	-

* p<0.05

** p<0.005; p<0.0001.

Similar to the previous model, among women with one or two children, those who did not complete elementary school were 2.5 times more likely (p = 0.038) to be sterilized compared to those who completed elementary school. Black women were almost five times more likely (p = 0.013) to have had the procedure among those with the same parity.

**Fig 2:**
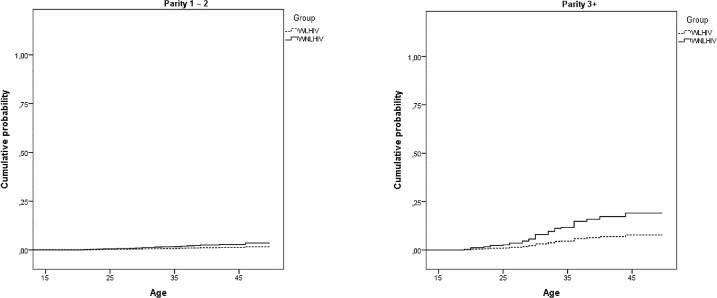
Cumulative probability of interval sterilization from models in Table 3 for women living and not living with HIV. São Paulo, 2013–14.

Fig 2 illustrates the cumulative probability of undergoing interval sterilization and indicates a lower probability than that estimated in the previous model, indicating that most sterilization happens at childbirth.

The third hazard model was estimated only for observations related to postpartum sterilization–including those carried out during a caesarean section and performed immediately after vaginal delivery (Table 4). The estimates indicate that WLHIV, in both parity-related groups, have a higher risk of being sterilized compared to WNLHIV. Similar to the previous two models, black women with lower parity were three times more likely (p = 0.006) to have had the procedure among those with the same parity, though schooling has not remained statistically significant.

**Table 4:** Hazard ratios for postpartum sterilizations by HIV status, education and color/race, and stratified by parity. São Paulo, 2013–14.

Characteristics	HR (IC95%)	
	1–2 children	3+ children
Group		
WLHIV	3.01 (1.64–5.51)***	2.36 (1.52–3.66)***
WNLHIV (ref)	-	-
Education		
Incomplete elementary school and lower	1.36 (0.72–2.54)	1.00 (0.60–1.69)
Complete elementary school and higher (ref)	-	-
Color/Race		
Brown (“parda”)	2.01 (0.94–4.28)	0.96 (0.57–1.60)
Black (“preta”)	3.38 (1.42–8.05)**	1.18 (0.58–2.42)
White (“branca”) (ref)	-	-

* p<0.05

** p<0.005; p<0.0001.

**Fig 3:**
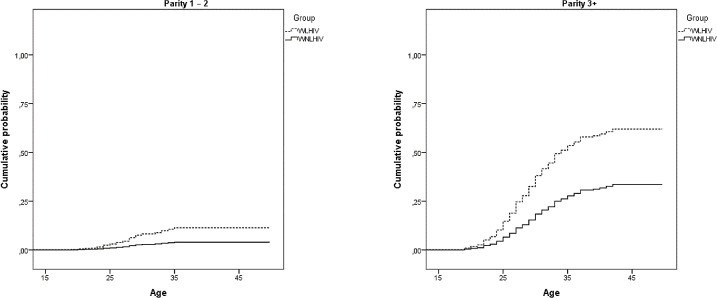
Cumulative probability of postpartum sterilization from the models in Table 4 for women living and not living with HIV. São Paulo, 2013–14.

The cumulative probability of having had a postpartum sterilization is much higher among women with three or more children regardless of HIV status, and increases with age (Fig 3).

The last model estimated the risk of undergoing a sterilization performed in conjunction with caesarean sections and indicates a higher risk among WLHIV (HR = 1.94; p = 0.012) compared to WNLHIV with lower parity (Table 5). No difference was observed among women with three or more children. There was also no significant difference in having sterilization when considering race/color and education in both parity-related groups.

**Table 5:** Hazard ratios for postpartum sterilization (performed during a caesarean section) by HIV status, education and color/race, and stratified by parity. São Paulo, 2013–14.

Characteristics	HR (IC95%)
	1–2 children	3+ children
Group		
WLHIV	1.94 (1.16–3.27)*	0.91 (0.6–1.38)
WNLHIV (ref)	-	-
Education		
Incomplete elementary school and lower	1.6 (0.94–2.72)	0.86 (0.53–1.4)
Complete elementary school and higher (ref)	-	-
Color/Race		
Brown (“parda”)	1.36 (0.73–2.53)	0.84 (0.53–1.33)
Black (“preta”)	1.99 (0.93–4.24)	1.32 (0.7–2.47)
White (“branca”) (ref)	-	-

* p<0.05

** p<0.005; p<0.0001.

**Fig 4:**
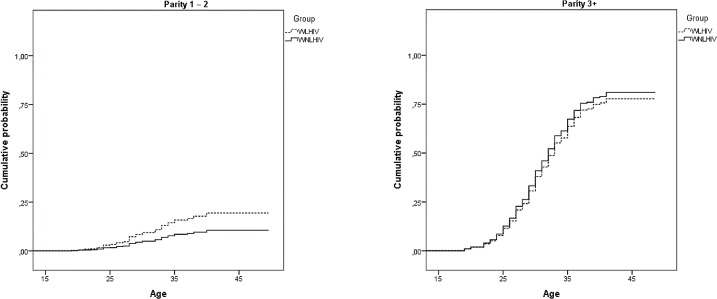
Cumulative probability of postpartum sterilization (performed during a caesarean section) from models in Table 5 for women living and not living with HIV. São Paulo, 2013–14.

Similar to the previous model, the cumulative probability of having had sterilization in conjunction with caesarean delivery is much higher among women with three or more children regardless of HIV status, and increases with age (Fig 4).

### Unmet demand for female sterilization

About a quarter of WLHIV and WNLHIV have reported that they would like to have been sterilized previously, with no significant difference between groups (Table 6). However, it is noteworthy that the proportion of unmet demand for sterilization is higher among younger WLHIV (AOR = 3.08; p = 0.0001) as compared to those aged 30 and over. No difference was observed among WNLHIV when considering age. Regarding parity, although the proportion of unmet demand for sterilization is higher among WNLHIV as well as WLHIV with three or more children compared to those with a lower parity, it is interesting to note that the proportion is significantly higher among those not living with HIV (AOR = 2.9; p = 0.000 versus AOR = 1.82; p = 0.018, respectively).

**Table 6:** Proportion of unmet demand for sterilization among WLHIV and WNLHIV according to age and parity and weighted binomial logistic regression. São Paulo, 2013–2014.

	Group					
	WLHIV			WNLHIV		
			Adjusted			Adjusted
	N	%	OR (95% CI)	N	%	OR (95% CI)
**Total**	**516**	**21.0%**	** **	**555**	**24.9%**	** **
Age at interview						
< = 29	58	39.9%	3.08 (1.67–5.67)***	184	23.2%	1.11 (0.74–1.67)
30+	458	18.5%	Ref	371	25.8%	ref
Parity						
1–2	399	18.9%	ref	440	20.6%	ref
3+	117	28.4%	1.82 (1.11–2.99)*	115	42.1%	2.9 (1.69–4.97)***

* p<0.05

** p<0.005

***p<0.0001.

Additionally, when asked about the best moment to be sterilized, the vast majority of women living with HIV (73.1%) clearly stated a desire for performing an interval sterilization, and as soon as possible (Table 7).

**Table 7:** Distribution of WLHIV and WNLHIV who want to be sterilized by preference regarding the moment to be sterilized. São Paulo, 2013–2014.

	Group	
	WLHIV	WNLHIV
	%	%
**Total**	**103**	**131**
at the next childbirth	11.6%	25.8%
as soon as possible, without going through childbirth to obtain sterilization	73.1%	53.3%
at another time, none of the above	15.3%	20.9%

## Discussion

Our study found no evidence of increased overall risk of sterilization among women living with HIV compared to those not living with HIV for women with lower and with higher parity. The prevalence of female sterilization performed after HIV diagnosis was similar to the observed among WNLHIV. The same conclusion was reached when the analysis took into account the time of exposure to the risk of sterilization and after adjusting for race/color and schooling level. However, significant differences regarding the risk of sterilization were observed depending on the timing and the type of sterilization procedure, as will be further discussed.

One previous study, conducted in 2003/04, identified evidence of a higher prevalence of female sterilization among women living with HIV compared to those not living with HIV [29]. However, this study only provided unadjusted prevalence of female sterilization carried out before and after HIV diagnosis, and more importantly, the results refers to a national convenience sample. These methodological and analytical differences make the comparison with our study very limited and difficult, since they could explain part of the divergent results.

Another possible explanation for the differences between the two findings would have been a decrease in the prevalence of sterilization among women living with HIV, similar to what was observed among the general female population in Brazil [16]. Several studies have argued whether part of this decline is due to a decrease in demand for sterilization or to the requirements imposed by the Family Planning Law between 1996 and 2000 to obtain the procedure [15;33]. In the case of WLHIV, it is also possible to assume that this decrease contains an additional element: it may be related to the advent of ART and the success of the most effective antiretroviral regimens for prophylactic prevention of mother-to-child transmission [23].

The advent of ART has turned AIDS into a chronic disease, and has offered the possibility of longer and healthier lives for women living with HIV. Advances in HIV treatment and prevention technologies have changed the context within which women decide whether or not to have children, and has thus expanded the realm of options for HIV-positive individuals, and in doing so, may have contributed to the decrease in the use of irreversible methods such as tubal ligation. In Brazil, reliable and widened access to HIV treatment and prevention concurred with the implementation of the Family Planning Law from 1996 to 2000 [14]. Our study was conducted ten years after the previous study and, therefore, could better reflect the changes in these contexts.

Similar to other studies carried out in Brazil among the general female population [13;16;32] and among women living with HIV [21], our study found that the probability of being sterilized is much higher among women with three or more children, regardless of HIV status.

Having had a child after HIV diagnosis has been pointed out as the most important factor associated with female sterilization [21]. In line with these results, we found a small proportion of sterilization among WLHIV who did not give birth after HIV diagnosis and a much higher proportion of sterilization among those who gave birth after diagnosis.

Apart from these results, by analyzing postpartum sterilizations separately from interval procedures, we were able to capture a much more complex scenario regarding the association between HIV infection and female sterilization, and provide new evidences regarding this relationship. The proportion of interval sterilization is significantly lower compared to postpartum procedures for women living and not living with HIV, which are in line with conclusions obtained by other studies [13;30;33]. However, its proportion is even lower for WLHIV compared to those not living with HIV. Same results were obtained when the model took into account parity and the time of exposure to the risk of sterilization, and was adjusted for race/color and schooling level. WLHIV with lower and higher parity tend to have a lower risk of undergoing interval sterilization compared to those not living with HIV. These results apparently indicate additional barriers in the access to services offering interval sterilization for these women.

An opposite scenario is observed regarding postpartum sterilization. There is strong evidence that the probability of being sterilized postpartum is significantly higher among WLHIV who gave birth after HIV diagnosis compared to WNLHIV. A study conducted in São Paulo in 1999/2000 also observed differences in the proportion of postpartum sterilization among HIV-positive women aged 18–49 who had received prenatal care in public health services, compared with a second sample drawn from the general population of women aged 18–40 who received prenatal care in both public and private prenatal clinics [30].

One could argue that women are at a higher risk of undergoing postpartum sterilization because they are also more at risk of having a caesarean delivery due to HIV infection. In our sample, 74% of the WLHIV who gave birth after diagnosis had a caesarean in the last delivery, compared to 42% among WNLHIV. However, by conducting a modeling only for women who gave birth through caesarean section, the risk of sterilization remains higher among WLHIV with less than three children.

Our results clearly demonstrate that part of the difference observed in the risk of obtaining the sterilization is due to a higher prevalence of caesarean sections among WLHIV. However, it is also evident that cesarean sections are not the sole factor that increases the risk of tubal ligation. Apparently, the higher probability of obtaining the procedure in conjunction with a caesarean section among WLHIV with lower parity indicates that access to tubal ligation is facilitated for these women. The existence of a less restrictive interpretation of the Family Planning Law with regard to women living with HIV could be a hypothesis to explain this phenomenon. According to a previous study, HIV infection would be perceived as a valid exemption from the prohibition on postpartum procedures by health care services and medical doctors [30].

The fact that we found no higher risk of being sterilized in conjunction with cesarean sections among WLHIV with three or more children deserves further discussion. One hypothesis is that sterilization and high parity are so intimately correlated that this procedure would be carried out either way among those women, regardless of their HIV status.

As for the association between color/race, education and sterilization, our study adds further aspects to the current discussion on that matter. By conducting an analysis stratified by parity, our findings suggest that color/race and years of schooling are not good predictors of the risk of female sterilization for women with higher parity for WLHIV as well as for WNLHIV. Black and less educated women have higher chances of being sterilized only among those with lower parity, in accordance to what was suggested among the general female population in 2006 [16]. The authors observed much higher proportion of sterilized women with less than two children or without reaching the ideal number of children among those with less education.

A study carried out among WLHIV in the Northeast region of Brazil in 2004 observed no evidence of association between female sterilization performed after HIV diagnosis and women´s educational attainment. However, as the model was adjusted for parity, we do not know the effects of race/color and education for different parity levels [21]

The discussion of whether color/race and education are good predictors for female sterilization in Brazil is far from over. The findings are very controversial and highly dependent on the period of time analyzed, the region of the country, the statistical analysis used and whether the outcome is related to interval or postpartum sterilization, or both [13;16;33].

In our study, when models analyze separately the moment of the procedure, the only remaining effect of education and color/race was observed for interval sterilization; being black and having lower level of educational attainment increases the probability of performing an interval sterilization among women with lower parity. On the other hand, no association was observed regarding sterilization performed in conjunction with caesarean section. A recent study, analyzing the risk of female sterilization among women who had experienced live births between 2001–2007, found that higher chances of being sterilized among black women are specific to interval sterilization in public hospitals [13]. Although our study does not provide information related to the health facilities where the delivery took place, black and lower schooling women usually have their births at public hospitals, due to financial restrictions.

The dramatic difference in access to postpartum and interval sterilization seems to be both unfair and very problematic to women in general, particularly to those living with HIV. There is a significant and similar unmet demand for sterilization in both groups; however, WLHIV want to have it done at a younger age and are much less willing to wait for the next childbirth or have another child in order to have access to sterilization: 73% would like to have it done as soon as possible. Nonetheless, WLHIV appear to be less likely to get interval sterilization than those not living with HIV. In our study, the lack of incentive to obtain tubal ligation on the part of health professionals was surprisingly higher in the group of WLHIV, 48% *versus* 27% among WNLHIV, which could hinder access to the procedure after childbirth.

In contrast to what occurs with sterilization carried out in conjunction with a cesarean section—for which there are several shortcuts that allow the bypassing of the Family Planning Law and which can be achieved through a direct agreement between the woman and the obstetrician in charge of the childbirth [20] -, having an interval sterilization within the public health care system depends on a referral from health services and professionals.

The great resistance to the promotion of dual protection, using both effective contraception and condoms, is part of the health care service scenario in which women living with HIV made their reproductive choices. This resistance makes it harder to obtain a referral for tubal ligation, which some women eventually undergo at the time of delivery after making a direct arrangement with the doctor. Health professionals are concerned that the use of other contraceptives will eventually cause the non-use or inconsistent use of condoms [18].

The occurrence of 70% of unintended pregnancies after HIV diagnosis observed in our study, questions the predominance of the use of condoms as the main contraceptive method, referred to as such by 69.8% of all sexually active WLHIV.

Some limitations of our study should be mentioned. First, we interviewed women in both groups who are users of public health care services. We do not have information regarding the reproductive choices of those who received care exclusively from private or insured health services. A previous study among the general female population suggests an increased access to postpartum sterilization when it is performed in conjunction with a caesarean section and when the childbirth occurs at private hospitals [13]. In our study, 25% of WNLHIV have had the procedure performed at private hospitals and only 10% of the sterilizations performed after HIV diagnosis were conducted at private hospitals. However, as the study does not provide information regarding the health facilities where the childbirths took place, it was not possible to assess the influence of the place of delivery in the risk of female sterilization. Another limitation is the small number of observations regarding interval sterilization among women with lower parity, which may lead to a diminished statistical power to detect significance differences between the two groups.

Despite this limitation, our study provides some important clues about how HIV infection and other factors may jointly influence women´s access to sterilization. At the same time, many questions remain, pointing to the need for further studies on the relationship between HIV infection and reproductive outcomes in other regions of Brazil and elsewhere.

## Final Remarks

In contrast to earlier studies, our analysis was able to estimate the risk of women getting sterilized after HIV diagnosis compared to those not infected with HIV. Moreover, by estimating separately the risk associated with interval and postpartum sterilization, we found that WLHIV have lower probability of undergoing interval sterilization but higher of undergoing postpartum sterilization compared to WNLHIV. It was also possible to demonstrate that the existence of an increased risk of postpartum sterilization for WLHIV cannot be credited exclusively to a higher prevalence of caesarean delivery in this group. Lastly, the results suggest that the impact HIV infection may have in the access to female sterilization during a caesarean section varies according to parity. Only WLHIV with one to two children have a higher probability of obtaining a procedure in conjunction with a caesarean in comparison to WNLHIV with the same parity.

There is no doubt that caesarean sections represent a major shortcut to having tubal ligation, which therefore raises some questions. The first question addresses the possibility of interpreting the highest probability of access to postpartum sterilization as a result of certain "facilitation" by health professionals in order to broaden access to the procedure for WLHIV.

On the one hand, it is questionable to what extent this “facilitation” would be an expression of a hidden prejudice (but still effective) in relation to WLHIV’s right to have (more) children. On the other hand, it could mean a greater solidarity of health professionals with women, who under the risks and uncertainties posed by their HIV status, could access the control of their reproductive capacity. In both cases, it reflects the possibility of making the laws more flexible by reinterpreting them; processes that are carried out at the institutional level by the agents involved in everyday health care.

However, a list of other issues is necessarily open to discussion. For example, this "shortcut" could again drive women into the position of interpersonal negotiations. In terms of reproductive health politics, this means that the process of being sterilized is no longer situated within the boundaries of the regulatory level and, it turns out to be mostly (again) a matter of “bargaining” between the woman and the health professional. This is exactly the existing situation before the Family Planning Law, and one of the reasons that led it to be approved.

The attempt of dissociating sterilization from caesarian section has not yet succeeded among the general female population, even twenty years after the approval of the family planning law, as shown by recent studies [13;15;17]. In this sense, our study adds further challenges to both national and local health authorities. Specific hurdles within HIV specialized health care services which limit access to interval sterilization must also be identified and addressed. At the same time, it is vital to review the legal restriction of postpartum sterilization in cases where an obstetrical indication of surgical delivery exists and women have passed through the legal process to decide and require a sterilization. Obstetrical indication of surgical delivery is particularly frequent among women living with HIV, therefore the revision of this restriction would avoid unnecessary risk of another surgical procedure to perform a sterilization.

Another key point is the urgent need to increase information and access to a wider range of contraceptive methods for WLHIV. Health policy makers at local and national levels should actively promote dual method protection strategies to target women living with HIV and also health professionals in order to overcome the barriers to effective contraception. Moreover, since condom use may decrease in the future in the context of the preventive effect of ART, promoting dual methods will expand the choices regarding the reproductive rights of women living with HIV [34].

Finally, it is crucial to have the involvement of WLHIV in the discussion of these and other issues related to their reproductive health. However, studies have indicated that the right and the possibility for WLHIV to choose from an entire range of contraceptive methods seem not yet to be endorsed by HIV-positive women themselves [34]. Thus, it is important to find ways to bring WLHIV to the core of the debate in order to reach a consensus on how to change standards and policies if we really want to ensure the reproductive and sexual rights of women living with HIV.

## Supporting Information

S1 File Genih data file. (XLSX)

## References

[1] UNAIDS. Prevention gap report. Geneva: UN Joint Programme on HIV/AIDS; 2016. http://www.unaids.org/sites/default/files/media_asset/2016-prevention-gap-report_en.pdf. Accessed on Sep 2016.

[2] Connor EM, Sperling RS, Gelber R, Kiselev P, Scott G, O'Sullivan MJ, et al Reduction of maternal-infant transmission of human immunodeficiency virus type 1 with zidovudine treatment. Pediatric AIDS Clinical Trials Group Protocol 076 Study Group. N Engl J Med. 1994;331(18): 1173–80. 10.1056/NEJM199411033311801 7935654

[3] Bedimo AL, Bessinger R, Kissinger P. Reproductive choices among HIV-positive women. Soc Sci Med. 1998;46(2): 171–9. 9447641 10.1016/s0277-9536(97)00157-3

[4] Figueroa-Damian R, Villagrana-Zesati R. Factors associated with acceptance of postpartum tubal ligation among HIV-infected women. Salud Publica Mex. 2001;43(2): 97–102. 11381847

[5] Thackway SV, Furner V, Mijch A, Cooper DA, Holland D, Martinez P, et al Fertility and reproductive choice in women with HIV-1 infection. AIDS. 1997;11(5): 663–67. 9108948 10.1097/00002030-199705000-00014

[6] van Benthem BH, de V I, Delmas MC, Larsen C, van den HA, Prins M. Pregnancies before and after HIV diagnosis in a European cohort of HIV-infected women. European Study on the Natural History of HIV Infection in Women. AIDS. 2000;14(14): 2171–78. 11061659 10.1097/00002030-200009290-00014

[7] Vincenzi I, Jadand C, Couturier E, Brunet JB, Gallais H, Gastaut JA, et al Pregnancy and contraception in a French cohort of HIV-infected women. SEROCO Study Group. AIDS. 1997;11(3): 333–38. 9147425 10.1097/00002030-199703110-00011

[8] Smits AK, Goergen CA, Delaney JA, Williamson C, Mundy LM, Fraser VJ. Contraceptive use and pregnancy decision making among women with HIV. AIDS Patient Care STDS. 1999;13(12): 739–46. 10.1089/apc.1999.13.739 10743537

[9] Healton C, Taylor S, Messeri P, Weinberg G, Bamji M. Effects of ZDV-based patient education on intentions toward ZDV use, HIV testing and reproduction among a US cohort of women. AIDS Care. 1999;11(6): 675–86. 10.1080/09540129947587 10716008

[10] Kline A, Strickler J, Kempf J. Factors associated with pregnancy and pregnancy resolution in HIV seropositive women. Soc Sci Med. 1995;40(11): 1539–47. 7667658 10.1016/0277-9536(94)00280-7

[11] IBGE. Censo Demográfico 2010: Resultados gerais da amostra Rio de Janeiro: Instituto Brasileiro de Geografia e Estatística; 2012.

[12] Cavenaghi S, Berquó S. Perfil socioeconômico e demográfico da fecundidade no Brasil de 2000 a 2010. In Cavenaghi S, Cabella W, editors. Comportamiento reproductivo y fecundidad en América Latina: Una agenda inconclusa; 2014. pp. 67–89.

[13] Amaral EFL, Potter JE. Determinants of female sterilization in Brazil, 2001–2007 Monica, CA: RAND Corporation; 2015.

[14] MS. Portaria SAS/MS-48, de 11/2/99 que Regulamenta a Lei Federal 9263. Brasília: MS; 1999.

[15] Caetano AJ. Esterilização cirúrgica feminina no Brasil, 2000 a 2006: aderência à lei de planejamento familiar e demanda frustrada. Revista Brasileira de Estudos de População. 2014;31(2): 309–31. Available: http://www.scielo.br/pdf/rbepop/v31n2/a05v31n2.pdf. Accessed on Dec 2015.

[16] Perpétuo IHO, Wong LR. Desigualdade socioeconômica na utilização de métodos anticoncepcionais no Brasil: uma análise comparativa com base nas PNDS 1996 e 2006. In: Ministério da Saúde, Centro Brasileiro de Análise e Planejamento (CEBRAP), editors. Pesquisa Nacional de Demografia e Saúde da Criança e da Mulher—PNDS 2006: Dimensões do processo reprodutivo e da saúde da criança; 2009. pp. 87–107.

[17] Domingues RMSM Dias MAB, Nakamura-Pereira M Torres JA, d'Orsi E Pereira APE, et al Processo de decisão pelo tipo de parto no Brasil: da preferência inicial das mulheres à via de parto final. Cad Saude Publica. 2014;30: S101–S116. Available: http://www.scielo.br/pdf/csp/v30s1/0102-311X-csp-30-s1-0101.pdf. Accessed on Feb 2016.

[18] Villela WV, Barbosa RM. Prevention of the heterosexual HIV infection among women: is it possible to think about strategies without considering their reproductive demands? Revista Brasileira de Epidemiologia. 2015;18: S131–S42. 10.1590/1809-4503201500050010 26630303

[19] Garcia S, Berquó E, Lopes F, Lima LP, Souza F. Práticas sexuais e vulnerabilidades ao HIV/aids no contexto brasileiro. Qualificando os números: estudos sobre saúde sexual e reprodutiva no Brasil. Demografia em debate Belo Horizonte: ABEP/UNFPA; 2008.

[20] Knauth DR, Barbosa RM, Hopkins K. Between personal wishes and medical "prescription": mode of delivery and post-partum sterilisation among women with HIV in Brazil. Reprod Health Matters. 2003;11(22): 113–21. 14708402 10.1016/s0968-8080(03)22100-5

[21] Oliveira F, Kerr L, Frota A, Nobrega A, Bruno Z, Leitao T, et al HIV-positive women in northeast Brazil: tubal sterilization, medical recommendation and reproductive rights. AIDS Care. 2007;19(10): 1258–65. 10.1080/09540120701405411 18071969

[22] Teixeira LB, Pilecco FB, Vigo A, Knauth DR. Sexual and reproductive health of women living with HIV in Southern Brazil. Cad Saude Publica. 2013;29(3): 609–20. Available: http://www.scielo.br/pdf/csp/v29n3/a18v29n3.pdf. Accessed on Jan 2016 23532295 10.1590/s0102-311x2013000300018

[23] Ministério da Saúde. Dados Epidemiológicos HIV AIDS. 2015;4(1).

[24] Santos NJS, Barbosa RM, Pinho AA, Villela WV, Tirza A, Filipe EMV. Contextos de vulnerabilidade para o HIV entre mulheres brasileiras. Cad. Saúde Pública 2009;25(Supl.2): S321–S33. 10.1590/s0102-311x2009001400014 19684939

[25] Ávila MB. Direitos sexuais e reprodutivos: desafios para as políticas de saúde. Cad. Saúde Pública 2003;19(Supl.2): S465–S469. Available: http://www.scielo.br/pdf/csp/v19s2/a27v19s2. Accessed on Feb 2016. 10.1590/s0102-311x2003000800027 15029365

[26] Menezes G, Aquino EML. Pesquisa sobre o aborto no Brasil: avanços e desafios para o campo da saúde coletiva. Cad. Saúde Pública. 2009;25(Supl.2): S193–S204. Available: http://www.scielo.br/pdf/csp/v25s2/02.pdf. Accessed on Feb 2016 10.1590/s0102-311x2009001400002 19684927

[27] Vieira EM. Políticas públicas e contracepção no Brasil In: Berquó E, editor. Sexo e Vida: Panorama de saúde reprodutiva no Brasil. Campinas: Editora da Unicamp; 2003 pp. 151–96.

[28] Vieira EM. Badiani R, Dal Fabbro AL, Rodrigues AL Jr. Characteristics of anticontraception methods used in Sao Paulo State, Brazil. Rev Saude Publica. 2002;36(3): 263–70. 12131963 10.1590/s0034-89102002000300002

[29] Barbosa RM, Pinho AA, Santos NS, Filipe E, Villela W, Aidar T. Aborto induzido entre mulheres em idade reprodutiva vivendo e não vivendo com HIV/aids no Brasil. Ciência & Saúde Coletiva. 2009;14(4):1085–99. 19721949 10.1590/s1413-81232009000400015

[30] Hopkins K. Maria BR, Riva KD, Potter JE. The impact of health care providers on female sterilization among HIV-positive women in Brazil. Soc Sci Med. 2005;61(3): 541–54. 10.1016/j.socscimed.2004.12.011 15899314

[31] Elder G., Johnson MK, Crosnoe R. The emergence and development of life course theory In: Mortimer J.T.; Shanahan M.J., *Handbook of the life course*. New York: Kluver academic/Plenum Publishers, 2003.

[32] Amorim F, Cavenaghi S, Alves J. Mudanças recentes no uso de métodos contraceptivos no Brasil e na Colômbia: com especial menção à esterilização masculina e feminina In: Wong LR, editor. Población y salud sexual y reproductiva en América Latina. Rio de Janeiro: Asociación Latinoamericana de Población; 2008 pp. 101–30.

[33] Potter JE, Perpetuo IH, Berquo E, Hopkins K, Leal OF, de Carvalho Formiga MC, et al Frustrated demand for postpartum female sterilization in Brazil. Contraception. 2003; 67(5): 385–90. 12742562 10.1016/s0010-7824(03)00039-8

[34] Maraux B, Hamelin C, Bajos N, Dray-Spira R, Spire B, Lert F. Women living with HIV still lack highly effective contraception: results from the ANRS VESPA2 study, France, 2011. Contraception. 2015;92(2): 160–69. Available: http://www.contraceptionjournal.org/article/S0010-7824(15)00178-X/pdf. Accessed on July 2015. 10.1016/j.contraception.2015.04.010 25940932

